# Pancreatic Cancer Microenvironment and Cellular Composition: Current Understandings and Therapeutic Approaches

**DOI:** 10.3390/cancers13195028

**Published:** 2021-10-08

**Authors:** Linh-Huyen Truong, Siim Pauklin

**Affiliations:** Botnar Research Centre, Nuffield Department of Orthopaedics, Rheumatology and Musculoskeletal Sciences, Old Road, University of Oxford, Oxford OX3 7LD, UK; linhhuyen.truong@ndorms.ox.ac.uk

**Keywords:** pancreatic ductal adenocarcinoma, cancer therapy, tumor microenvironment, fibroblasts, immune cells, tumor stroma

## Abstract

**Simple Summary:**

There is currently no effective treatment for Pancreatic Ductal Adenocarcinoma (PDAC), and it is still the deadliest cancer, despite great research efforts in recent decades. The complexity in the tumor microenvironment (TME) is one of the main roots for the refractory nature of PDAC to treatment. Components of the PDAC TME are complex, dynamic, and closely reciprocate with each other from the early stages of pre-neoplastic lesion and during tumor progression. Detailed insight of the PDAC tumor microenvironment is needed for developing combined therapeutics that would target cancer cells and their supportive milieu for more efficient PDAC treatment.

**Abstract:**

Pancreatic ductal adenocarcinoma (PDAC) remains one of the most lethal human solid tumors, despite great efforts in improving therapeutics over the past few decades. In PDAC, the distinct characteristic of the tumor microenvironment (TME) is the main barrier for developing effective treatments. PDAC TME is characterized by a dense stroma, cancer-associated fibroblasts, and immune cells populations that crosstalk to the subpopulations of neoplastic cells that include cancer stem cells (CSCs). The heterogeneity in TME is also exhibited in the diversity and dynamics of acellular components, including the Extracellular matrix (ECM), cytokines, growth factors, and secreted ligands to signaling pathways. These contribute to drug resistance, metastasis, and relapse in PDAC. However, clinical trials targeting TME components have often reported unexpected results and still have not benefited patients. The failures in those trials and various efforts to understand the PDAC biology demonstrate the highly heterogeneous and multi-faceted TME compositions and the complexity of their interplay within TME. Hence, further functional and mechanistic insight is needed. In this review, we will present a current understanding of PDAC biology with a focus on the heterogeneity in TME and crosstalk among its components. We also discuss clinical challenges and the arising therapeutic opportunities in PDAC research.

## 1. Introduction

Pancreatic cancer is the most lethal human malignancy, and there has been no major improvement in the therapeutic progress against this disease in the last 40 years [1,2]. Although this cancer accounts for only 3% of newly recorded cancer cases, it is ranked as the fourth leading cause of cancer-related death worldwide, and it is predicted to take second place in the U.S. by 2030 [1,3]. GLOBOCAN estimated data for pancreatic cancer present a globally increasing incidence with a closely similar mortality with 495,773 new diagnosed cases and 466,003 related deaths in 2020 [4]. In contrast to the downward trend of almost all cancers, pancreatic cancer is on the rise with new cases and new deaths increasing by 0.4% and 0.3% each year, respectively [5]. Overall, the current burden of this deadly cancer reflects the main risk factors, age, obesity, diabetes, pancreatitis, and its characteristics, which limit early diagnosis and treatment options [6].

The pancreatic ductal adenocarcinoma (PDAC) is derived from ductal epithelial cells and is the most common malignancy of the exocrine pancreas, which is responsible for more than 90% of pancreatic cancer cases [1]. To date, surgical resection is the only curative treatment, but this is only eligible to patients diagnosed at an early state, which account for 11% of all cases [5,7]. Systemic therapy is the mainstay treatment option for almost all PDAC patients, including the resectable group [7]. Unfortunately, despite huge efforts in research, PDAC still shows an extremely poor prognosis, and tumor relapse occurs in many post-surgical patients [2]. This dismal scenario of PDAC patients is caused by the late diagnosis, aggressive disease progress with early metastases, tumor complexities, and inefficient therapeutics.

Currently, there is no validated test for early detection of pancreatic cancer, and almost all patients are asymptomatic at early stages, while nearly 90% of this cancer are diagnosed at advanced stages [4,8]. As a disease of the elderly with a median age of 70 years at diagnosis [5], symptoms at the early stage of the malignancy, such as aches and unexplained weight loss, are non-specific and, in most cases, remain unnoticed [8]. The clinical manifestation of these early stages usually cannot be monitored by imaging because the pancreas is too deep to palpate [2]. Furthermore, there is no validated genetic test for PDAC due to the absence of specific and sensitive biomarkers [4]. Therefore, symptoms at diagnosis, such as diabetes, jaundice, and thrombosis, mark already advanced disease stages with a deteriorated pancreas in almost all patients [7].

Pancreatic cancer is a multifactorial and very aggressive disease. The location in the retro-peritoneum is surrounded by many blood vessels, and the ability of tumors to promote vasculogenesis allows cancer cells to metastasize microscopically at a very early stage. Therefore, undetectable residual tumor material remains in a patient even in a complete surgical resection, which causes tumor relapse. Pancreatic cancer also manifests as a syndrome with systemic comorbidities, such as hyper catabolic state of cachexia and muscle wasting, which negatively influence the patients’ quality of life [9]. Besides systemic comorbidities, the pressing of the tumor on the perineural surrounding, the spine, and organs in the abdomen make PDAC one of the most painful cancers. Together with unrelated ailments typical in the aged patients, many PDAC patients suffer from an overall poor health condition and, therefore, are not eligible for receiving treatment therapies.

The heterogeneity of the pancreatic tumor contributes to its unique characteristics. At the cellular level, PDAC mostly arises from pancreatic intraepithelial neoplasia (PanIN) or intra-ductal papillary mucinous neoplasms (IPMNs) and mucinous cystic neoplasms [8,10]. In the pre-neoplastic lesions, cells accumulate genetic and epigenetic alterations in oncogenic genes and trigger aberrant signaling cascades, which promote tumorigenesis and metastasis [11]. These molecular alterations also influence the metabolic reprogramming within the tumor microenvironment (TME) [11,12]. As an inflammation-driven cancer, PDAC is associated with pancreatitis, and the TME has a high level of heterogeneity in both cellular and acellular components [13]. In current treatment strategies for PDAC patients, its stroma-rich TME limits the access of systemic therapies to cancer cells and contributes to poor clinical outcomes [13,14]. In this review, we will summarize new insights on the TME biology of pancreatic cancer and discuss the translational strategies.

## 2. Heterogeneity in PDAC

### 2.1. Intra-Tumor Heterogeneity

Pancreatic cancer is highly heterogeneous at morphological, cellular, and molecular level. The histological, genetic, transcriptomic, and metabolic features of PDAC can be used to classify the cancer into subtypes associated with different clinical outcomes and therapeutic responses. Moreover, the intratumor heterogeneity includes the clonal evolution of cancerous cells, differentiation and dedifferentiation of cell identity, and a complicated crosstalk between cellular and acellular compartments in the peri-tumor microenvironment [15].

### 2.2. Histological Heterogeneity

Pancreatic cancers have heterogeneous histological features partly due to the different cell types from which the tumors originate. The normal pancreas is a gland with dual functions, consisting of cells with exocrine function that secrete digestive juices through ducts, and endocrine cells that secrete hormones into the blood. Most pancreatic cancers occur in the exocrine compartment, while a small minority, neuroendocrine tumors, form in the endocrine part. The majority of malignant epithelial tumors are ductal adenocarcinoma that can be further divided into adenosquamous, colloid, medullary, hepatoid, signet ring, undifferentiated carcinomas, and undifferentiated carcinomas with osteoclast-like giant cells [16]. Acinar cell carcinoma, pancretoblastoma, and solid pseudopapillary neoplasm are less common types of exocrine cancers. Although adenosquamous carcinoma has been reported as the most aggressive subtype with poor prognosis [17], the molecular features of the histological subtypes of pancreatic cancers are still under investigation.

### 2.3. Cancer Cell Heterogeneity

Conventionally, the intra-tumor cellular heterogeneity mostly refers to the molecular differences related to genomic instability, individual mutations, copy number alterations, and expression level of oncogenes and tumor suppressor genes [18,19]. Those genetic patterns represent a clonal evolution during tumor progression from the very first pre-neoplastic events [19]. The activating mutation in KRAS is present in >95% PDACs, and it is detected already in very early neoplastic precursor lesions [20]. Activating KRAS signaling is accompanied by certain inactivating mutations in tumor suppressor genes, such as TP53, CDKN2A (p16), and SMAD4, which occur in 30–70% of pancreatic cancers [20,21]. In addition to point mutations in particular genes that contribute to disease pathophysiology, the copy number alteration commonly involves homozygous insertions or deletions [20]. Structural variations on the chromosome have characterized pancreatic cancer subtypes at the genomic level as stable, locally rearranged, scattered, and unstable subtypes [20,22]. Besides genomic heterogeneity, epigenetics alters the transcriptome and contributes to sub-clonal variation in PDAC by DNA hypo- or hypermethylation, histone modifications, and non-coding RNA molecules [23]. The epigenetic regulators that are found deregulated in PDAC are methyltransferases (MLL2, SETD3, KAT2A), enhancer of zeste homolog 2 (EZH2), and histone deacetylase (HDAC1C) [23]. The enzyme EZH2 is the catalytic subunit of polycomb repressive complex 2 (PRC2) that can repress the expression of downstream target genes by trimethylation of Lysine 27 in histone 3 (H3K27me3). The change in the enzymatic activity of epigenetic regulatory enzymes leads to deregulated deposition of activating (e.g., H3K4me3, histone acetylation) and repressive histone (e.g., H3K27me3, histone deacetylation) or DNA marks (cytosine methylation) marks on promoters and enhancer sequences. These epigenetic changes often lead to the repression of tumor suppressor genes, cell cycle inhibitors, and apoptosis inducers, whereas genes involved in promoting cell growth, cell cycle, and cell survival are upregulated. Alongside genetic changes in precursor lesions, several miRNAs are involved in oncogene activation and tumor suppressor gene inactivation that lead to perpetual growth of malignant cells over time [23,24]. The genetic and epigenetic alterations not only foster the cancerous transformation via deregulating oncogenes and tumor suppressor genes but also via activating the stem-like characteristics which usually are only present in normal stem cells or progenitor cells [25,26]. Understanding the heterogeneity of cancer cells, along with the dedifferentiation process of cell identity and the characteristics of cancer stem cells (CSCs), will provide insight to therapeutic resistance and unveil novel therapeutic avenues in PDAC.

## 3. Cells in Tumor Microenvironment

**Figure 1:**
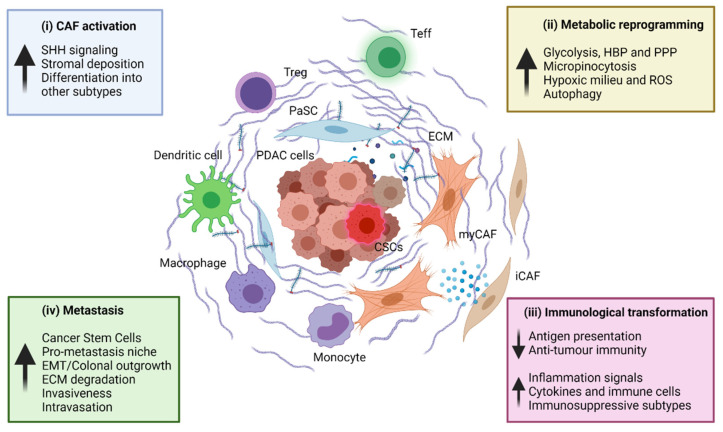
PDAC microenvironment and the cellular interplay. Pancreatic ductal adenocarcinoma (PDAC) is highly heterogeneous in both cellular and acellular components. The tumor cells themselves are varied in mutation/gene expression profile, which manipulates the surrounding fibroblasts and immune cells. In turn, the tumor microenvironment components interact with each other and with tumor cells in a pro-tumor support fashion. The major interplay in PDAC includes (**i**) CAFs activation which leads to creating a desmoplastic stroma, (**ii**) metabolic reprogramming, (**iii**) immunological transformation, and (**iv**) metastasis initiating events in early stages of tumor development. Treg = regulatory T cell, Teff = effector T cell, PaSC = Pancreatic stellate cells, PDAC = pancreatic ductal adenocarcinoma, EMC = extracellular matrix, myCAF = myofibroplastic cancer associated fibroblast, iCAF = inflammatory cancer associated fibroblast. Figure made in BioRender.

The heterogeneity of PDAC not only comes from the intra-tumor cellular components but also from the dynamic TME with highly diverse cellular and extracellular components (Figure 1). PDAC is described as harboring a desmoplastic stroma rich in cancer-associated fibroblasts (CAFs), infiltrated by the immune compartments and their soluble functional molecules in the TME [27,28]. Novel therapies based on preclinical data can lead to divergent effects in clinical trials because cross-talk between cancer cells and cellular TME components is still insufficiently understood. Therefore, delineating the biological and functional aspects of TME are of great interest for potential therapeutic options.

## 4. Cancer-Associated Fibroblasts in PDAC

Fibroblasts are conventionally characterized by the expression of the fibroblast activation protein (FAP) and α-smooth muscle actin (α-SMA), and these cells are responsible for secreting ECM proteins during the desmoplastic response of the wound repair process [29]. The healthy pancreas contains a rare population of resident fibroblasts called pancreatic stellate cells (PaSCs), which are bone marrow mesenchymal stem cell (BM-MSC)-derived and play a critical function in providing a homeostatic response to tissue injuries caused by pancreatitis or partial pancreatectomy [30,31]. CAFs in pancreatic neoplasm function as a tumor-promoting component, although they are a diverse cell population.

The normal functions of PaSCs are hijacked in PDAC formation to support tumor growth, immune invasion, and the metastatic process, as well as resistance to therapeutics [32,33]. The α-SMA+ CAFs are responsible for the secretion of EMC and soluble molecules which create a TME that benefits tumor growth and also provides a rigorous physical barrier to therapeutic agents [34,35]. The in vitro co-culture of PDAC cells with PaSCs increases cell proliferation and migration of malignant cells [36], while the co-injection of PaSCs with PDAC cells in mouse increases tumor size and liver metastasis compared to PDAC cells alone [37,38]. Contrary to the thorough preclinical studies and a clear molecular and cellular mechanism, the therapeutic combinations which directly target α-SMA+ CAFs, chimeric antigen receptor T-cell (CAR-T) therapy, or small inhibitor targeting FAP+ cells, have failed in the clinical trial and presented unexpected clinical outcomes [39,40,41]. This observation may be explained by the fact that stroma cells naturally restrain tumor growth but are recruited and re-programmed during neoplastic processes to tumor supporting cells. This suggest that the CAF-targeting therapy needs to be given at a right time point of cancer development.

Genome-wide studies have revealed distinct subtypes of CAFs in PDAC which provide a more precise model of CAF function in both anti-tumorigenic and pro-tumorigenic effects. The α-SMA+ CAFs are defined as myofibroblastic CAFs (myCAFs) which locate close to the tumor cell and resemble the activation of PaSCs in the normal pancreas [42]. They depend on TGF-β signaling, produce extracellular matrix components, and are responsible for stroma deposition [37,42]. The cancer-promoting function might belong to the inflammatory CAFs (iCAFs) which are located further away from tumor cells. iCAFs are characterized by the expression of CXCL12 and IL-6, and they act as PaSCs in their immune activation stage via NF-kB signaling [42]. The reprograming of CAFs into the CXCL12+ IL-6+ phenotype is induced by IL-1α via the activation of JAK-STAT3 signaling [43]. iCAFs also produce inflammatory cytokines Leukemia Inhibitory Factor (LIF) and IL-6 in response to paracrine signals from malignant cells and promote immune invasion, as well aschemo-resistance, and, ultimately, worsen the overall survival of PDAC patients [43]. The concentration of IL-6 in patient serum has been shown to correlate with metastasis to the liver and form a niche for PDAC liver metastases by inducing STAT3 signaling in hepatocytes [43].

Recently, novel CAF subtypes with distinct phenotypes have been identified in PDAC. One subtype is the antigen-presenting CAFs (apCAFs) which have a high expression of MHC-II and CD25 but low level of costimulatory genes CD40, CD80, CD86 [44]. While MHC-II is essential to interact with CD4+ T cells, the lack of costimulatory molecules needed for CD4+ T cell clonal proliferation may inhibit T cell-mediated anti-tumor immunity [45]. Additionally, in the PDAC-derived CAFs cultures, Meghna Waghray and colleagues identified a CAF population that expressed mesenchymal stem cell markers (CD44, CD49α, CD90, CD73) and account for an average of 6.9% of total ex vivo CAFs, and a portion of those mesenchymal stem cell CAFs (mscCAFs) was dynamic during the culture process compared with its fragment in the freshly dissociated tumor [46]. mscCAFs (or cancer-associated MSC of carcinogenesis- associated MSC (CA-MSC)) have a tumor-promoting effect by inducing polarization of macrophages in TME toward an immunosuppressive subtype [46,47].

Notably, CAF subtypes show a dynamic cellular identity and are able to interconvert at least in the ex vivo model. Changing culture conditions from an organoid-conditioned medium into monolayer culture also shifts iCAFs and apCAFs into myCAF phenotype [42]. In a mouse model of PDAC treated with AZD1480, a JAK inhibitor, iCAFs were reprogrammed to express myCAF features, therefore increasing the ECM deposition and restraining tumor growth [43]. This CAF plasticity also emphasizes the heterogeneity and diverse function of CAFs in pancreatic cancer which closely coordinate the neoplastic and immunological compartment in TME.

## 5. Tumor-Infiltrating Immune Cells

The immunomodulatory activity of immune cells is evident by their infiltration and accumulation during PDAC neoplastic progression. Low mutational burden and immunosuppressive pathways are usually promising signs in PDAC and indicate a strategy for an immunotherapeutic approach to treat PDAC. However, this represents challenges due to the complicated crosstalk between TME compartments within the tumor [48,49].

### Antigen Specific T Cells

Cancer formation is accompanied by anti-tumor immunity with the help from antigen specific T cells. Therefore, PDACs evolve processes to avoid immune recognition. The absence of anti-tumor immunity in PDAC is partly caused by the lack of antigen-presenting to the immune system. Therefore, this feature is exploited to develop vaccine therapies using cancer antigens or the adoptive T cell transferring of expanded tumor-T cell-specific clones [13]. Multiple vaccination strategies have been developed to target the PDAC-associated antigens, which include telomerase, KRAS, gastrin, carcinoembryonic antigen (CEA), MUC1, and mesothelin [50,51,52,53,54,55,56]. Notable vaccines have been well-established and evaluated to show promising enhancement of antigen-specific immunological responses in early phase trials. These strategies have aimed to compensate for the appropriate antigen-presenting activity in PDACs but have not provided much clinical significance because of the presence of several immunosuppressive pathways [55,57].

## 6. Immunosuppressive Cells

The infiltration of immunosuppressive cells can be detected at the very early lesions in PDAC development, while T effector cells are rare in TME and create an imbalance of pro-tumorigenic over anti-tumorigenic activity [48]. In PDAC, myeloid-derived suppressor cells (MDSCs), tumor-associated macrophages (TAMs), and regulatory T cells (Tregs) contribute to establishing an immunosuppressive TME [48].

### 6.1. Myeloid-Derived Suppressor Cells (MDSCs)

In the genetically engineered mouse model (GEMM) and orthotopic mouse model of PDAC, the most abundant immune cells are myeloid cells that are comprised of MDSCs and TAMs accounting for 5–10% and 15–20% of total tumor mass, respectively [58,59]. Suppressive myeloid cells are classified as MDSCs which express CD11b and Gr1 in mice and CD11b and CD33 in humans, while TAMs are CD11b low F40/80+ Gr1- in mice and HLA-DR+ and CD68+ in humans [60,61]. These myeloid cells are recruited into TME and polarize to a suppressive phenotype due to the secreted factors from tumor cells [62]. The infiltration of MDSCs and TAMs in the TME correlates to cancer prognosis [58].

### 6.2. Tumor-Associated Macrophages (TAMs)

Similarly to MDSCs, TAM infiltration is observed in KRAS-driven PDAC mouse model and is involved in carcinogenesis by their secretion of TNF, RANTES, and MMP9 [59,63,64]. In the early lesion, PanIN modulates macrophage function in TME via IL-13 and polarizes them from M1 to M2 subtype which plays an immunosuppressive function and promotes tumor growth [65]. Moreover, the CSF-1 in the TME also polarizes macrophages toward the M2 subtype, while GM-SCF shifts them toward the inflammatory M1 subtype [61]. TAMs foster cancer invasiveness by stimulating angiogenesis and inhibit anti-tumor responses of NK and T effector cells by expressing non-classical MHC-1 (HLA-G) and ligands of co-inhibitory receptor PD-1 and CTLA-4 [66]. Macrophage-induced IL-6 also promotes malignant progression via the JAK-STAT3 signaling pathway in early lesions [67].

### 6.3. Tumor-Associated Neutrophils (TANs)

The accumulation of tumor-associated neutrophils has been reported in PanINs, although their precise impact on carcinogenesis is not yet clear [68]. Under a pro-inflammatory condition, neutrophils are recruited into PDAC TME in response to TNF-α and IL-12 secreted by cancer cells and then re-programmed to play dual roles [68,69]. Upon TGF-β signaling, TANs are polarized into the N1 subtype which restrains cancer growth by recruiting and activating CD8+ T cells through releasing TNF-α, CCL-3, CXCL-9, and CXCL-10 [69]. In a different manner, Treg-derived IL-35 induces N2 neutrophils which play a pro-carcinogenic function by a variety of secreted molecules [70]. N2 neutrophil-derived reactive oxygen species (ROS), reactive nitrogen species (RNS), and neutrophil elastase (NE) can facilitate carcinogenesis and promote cancer development [71,72]. Neutrophils also produce hepatocyte growth factor (HGF) and proteolytic matrix metalloproteinases (MMPs), which support angiogenesis, tumor invasion, and metastasis [73,74].

### 6.4. Regulatory T Cells (Tregs)

Tregs are defined as CD4+Foxp3+ and play an important role in maintaining immunological self-tolerance in a normal context but suppress anti-tumor immunity in cancer. Several key mechanisms of Tregs have been proposed which lead to suppression of anti-tumor responses, including competition for ligands, the secretion of immune-suppressive cytokines, direct cytotoxicity, and the generation of inhibitory metabolites [75,76,77]. However, the precise mechanism of the immunomodulatory functions of Tregs in PDAC remains unclear. Tregs are detected in the TME during the neoplastic development from pre-neoplastic lesion to the invasive stages of the disease in which the accumulation and high level of Tregs are associated with poor prognosis and, ultimately, by a low survival rate in PDAC patients [78]. PDAC intratumor Tregs express a higher level of CTLA-4 and PD-1 than Tregs in pancreatic lymph nodes (Pan LNs) and peripheral inguinal lymph nodes (iLNs) [79]. Co-depletion of Tregs with either CD4+ or CD8+ T cells have been tested in GEMM, in which, Tregs present a pro-carcinogenic effect through their interaction with tumor-associated dendritic cells (CD11+ DC) [79]. Prolonged binding of intra-tumor Tregs and CD11+ DC does not impact CD4+ activation but CD8+ activation, together with a reduction of IFN-γ, CD44, and Granzyme B, reduction in tumor size and increased overall survival time [79]. Another study demonstrated that Treg depletion induces myeloid cell recruitment into TME via a CCR1-dependent pathway, which then suppresses anti-tumor immunity [80]. Additionally, Tregs show potential plasticity toward Th17 (Foxp3+RORγ+) and induce the production of IL-17, IL-23, and TGF-β which promote cancer growth and evasion [80]. However, another study found that Treg depletion resulted in (i) increasing chemoattractants to myeloid cells (CCL3, CCL6, CCL8) and (ii) immune-suppression genes in fibroblast (PD-L1, Arg1) [81]. In that way, Treg depletion reprograms myCAFs to an inflammatory phenotype, and the arising iCAFs have a pro-carcinogenic function [81]. In short, Tregs have a complicated crosstalk to CAFs and immune compartments in TME, which can result in both pro- and anti-tumor growth in the context of a desmoplastic cancer, such as PDAC.

### 6.5. Other T Cells Subsets

Recently, an increasing number of studies have revealed biological interactions of other infiltrated lymphocyte subpopulations within TME which may open new approaches for developing more effective therapies. The accumulation of γδT cells, CD4+ T helper cells, and B cells has been proposed to contribute to PDAC carcinogenesis, depending on the subtype they belong to. While the Th1-polarized CD4+ T cells (Th1), together with αβT cells (CD8+ T cells or cytotoxic T lymphocyte- CTL), restrain tumor growth, PDAC TME immune landscape seems to skew toward Th2 T helper cells and γδT cells which might foster tumor progression [82]. Th2 T cell secretes a broad range of cytokines, including IL-3, IL-4, IL-5, IL-6, IL10, IL-13, which promote pancreatic carcinogenesis by (i) increasing ECM deposition and collagen synthesis and (ii) promoting M2-polarization in macrophages [83,84]. Meanwhile, the γδT cells are normally absent in pancreatic tissue but increase dramatically and account for up to 75% of total T lymphocytes in PDAC. They locate in proximity to PSCs and induce stromal-derived IL-6 production, while resembling Foxp3+ Tregs in an immunosuppressive manner [85]. Indeed, PDAC infiltrated γδT cells express a higher level of galectin-9 (LGALS9) and PD-L1 (CD247) as compared to splenic γδT cells and tumor cells [85,86]. Those cells function as regulators of DC and CTL activation and, therefore, shape the adaptive anti-tumor immunity in PDAC TME [85]. The contribution of B cells in PDAC has remained controversial and were suggested to play dual functions in cancer progression. Several B cell depletion models have been used to demonstrate that intrapancreatic B cells accumulating during early PanIN might partly be attributed to regulatory B cell (Breg) biology, which may alter other immune cells rather than tumor cells [87]. In PDAC, infiltrated B cells induce immunosuppression by secreting IL-35, IL-10, and TGF-β and also influence stromal response in remodeling ECM by producing pro-fibrotic molecules PDGF-B and LOXL2 [88]. However, other studies reported that the IL10+ Bregs seem to be a minority in comparison to the whole infiltrated B cell population in PDAC TME but might accumulate in the tertiary lymphoid tissue [87,89]. Taken together, the immune landscape and the crosstalk between cellular populations within TME has remained to be established for potential immunotherapy approaches.

## 7. Acellular Component of Tumor Microenvironment

### 7.1. Extracellular Matrix and Structural Proteins

In PDAC, the secretion of various proteins into the surrounding microenvironment forms an extracellular matrix (ECM) with complex properties. The ECM consists of non-cellular mix of proteins, glycoproteins, proteoglycans, and polysaccharides and plays an important role in pancreatic cancer progression. These acellular components, including structural ECM proteins and soluble signaling molecules, play an important role in the crosstalk with cellular compartments. ECM provides a physical scaffold for its surrounding cells, binds growth factors, and regulates cell behavior. ECM can be divided into basement membrane that supports epithelial/endothelial cell features and interstitial matrix that supports the underlying stromal compartment [90]. The major components of the basement membrane are the network-forming collagens, such as type IV and type VIII collagen, whereas the interstitial matrix consists primarily of the fibrillar-forming collagens type I, II, III, V, XI, XXIV, XXVII and the beaded filament type VI collagen synthesized by the fibroblasts in the stroma. Cancer associated fibroblasts are key players in orchestrating the tumor microenvironment composition and tissue microarchitecture leading to excessive collagen deposition. The collagens in TME are often crosslinked and linearized leading to increased stiffening of the PDAC tissue [90]. This elicits behavioral effects on surrounding cancer cells and regulates cell proliferation, differentiation, gene expression, migration, invasion, metastasis, and survival [91]. Collagen deposition is also relevant for PDAC prognosis, since highly aligned stromal collagen is a negative prognostic factor after PDAC resection [92]. Mass spectrometric studies from human PDAC samples and GEMMs show that collagen types I and III are the main structural proteins of the ECM in PDAC, accounting for 90%, followed by type IV collagen and Hyaluronic acid (HA) [93]. A study by Koenig et al., demonstrated that collagen type I (COL1A) induces PDAC progression and metastasis by disrupting the E-cadherin-mediated cell-cell contact and up-regulating N-cadherins [93]. The neoplastic progression is also mediated by particular MMPs, such as MMP2 and MMP7, that construct collagen networks [94]. While collagen gives TME stiffness, HA is a megadalton glycosaminoglycan that has the ability to retain water and, thereby, increase interstitial fluid pressure and compress blood vessels within the tumor [95]. Besides these proteins, the secreted protein acidic and rich in cysteine (SPARC) shows increased expression in cancer samples and correlates with negative prognosis and treatment response [96,97].

Fibronectin is another important component of PDAC TME. It is a large multidomain glycoprotein dimer assembled by cell-driven forces into a fibrillar array that provides a scaffold for the deposition of other matrix proteins and binding sites for soluble factors in the TME [98]. Different cellular FN isoforms are produced by alternative splicing, and these have varying receptor binding ability and spatiotemporal expression. FN regulates the proliferation, motile behavior and fate of multiple cell types, largely through mechanisms that involve integrin-mediated signaling. FN expression during tumorigenesis can support proliferative signaling, promote angiogenesis, facilitate invasion and metastasis, modulate growth suppressor activity, and regulate anti-tumoral immunity [98]. Fibronectin also regulates the chemoresistance. For instance, FN secreted by pancreatic stellate cells in the ECM has a central role in the development of resistance to gemcitabine via activating ERK1/2 [99]. Therefore, fibronectin-blocking agents added to gemcitabine-based chemotherapy might counteract chemoresistance in PDAC and provide better clinical outcomes.

### 7.2. Secreted Signalling Molecules

In addition to the abundance of structural ECM proteins, PDAC is also rife with signaling molecules secreted by subpopulations of cells in the TME, typically TGF-β or pro-inflammatory cytokines, such as IL-6, IL-1, and TNF-α. TGF-β signaling is a pleiotropic pathway with a function that is dependent on the cellular context and gene mutations [100]. Research in PDAC mouse models indicates that the tumor-intrinsic TGF-β plays a tumor suppressive function in the early stages through SMAD4-regulated genes, but the global impact of stromal-derived TGF-β promotes cancer growth and immunosuppressive mechanisms in the later stages, particularly upon SMAD4 inactivation [101]. The enhanced expression of TGF-β or TGF-β receptor complex (TGF-βRI/II) is found in nearly half of PDAC tumors and is associated with poor prognosis [102]. In the GEMM model, global depletion of TGF-βRII confines PDAC growth by inhibiting stromal fibrosis and restores anti-tumor immunity [103]. Although the precise mechanism remains unknown, the experimental evidence emphasizes the critical role of TGF-β signaling in PDACs and its importance as a therapeutic target in clinical trials. Inflammation is a hallmark in PDAC; therefore, chemokines and pre-inflammatory cytokines secreted by CAFs and infiltrated immune cells are also abundant in PDAC TME. These contribute significantly on the complexity and dynamics of TME and are associated with tumor progression.

## 8. Cellular Interplay in PDAC

### 8.1. CAF Activation

The expression of sonic hedgehog (SHH) signaling is elevated from the very early stages of PanINs but absent in normal pancreatic tissue, suggesting a role throughout tumor progression [104]. SHH expression in PDAC cells is upregulated by KRas mutation via NF-kB signaling and it functions both via autocrine manner to support cancer cells themselves and by impacting the surrounding cells via a paracrine mechanism [104]. One of the first and influential effects of SHH signaling is to activate PSCs into the activated myCAFs stage which express FAP and alpha-smooth muscle protein. Steele et al. recently demonstrated that the hedgehog pathway is a key factor for activating and maintaining myCAFs in KPC tumors, rather than other CAF subtypes [105]. In turn, myCAFs deposit ECMs to establish the TME scaffold and support cancer growth [104,105]. The SHH signaling activity also affects the ratio of myCAFs/iCAFs in PDAC tumors [105]. Therefore, it influences the expression of inflammatory signaling molecules from the iCAFs population and, thereby, affects the immune cell infiltration profile in TME. Although there is accumulating evidence of SHH biological function in PDAC, the failure of therapeutic targeting of SHH signaling underlines the requirement for deciphering the precise mechanisms of SHH function in PDAC.

### 8.2. Metabolic Reprogramming

In PDAC, tumor cells need a strategy to grow in a nutrient-deficient environment caused by dense stroma and poor perfusion. Thus, genetic alteration, together with dynamic signaling in TME, alters the metabolism of PDAC cells to provide an adequate source of materials for cancer cell growth. In KRAS-driven PDAC, the glucose uptake and glycolysis are strongly enhanced by increased expression of the glucose transporter GLUT1 and multiple glycolysis enzymes, such as HK1/2, PFK1, and LDHA [106]. Increased glycolysis is enriched by the hypoxic state induced by the hypovascular nature of PDAC through the hypoxia-inducible factor (HIF) pathway [106]. Not only glycolysis but other glucose utilization pathways, such as hexosamine biosynthesis (HBP) or non-oxidative arm of pentose phosphate (PPP), are also enhanced to generate materials to promote cell proliferation [107]. In addition, glutamine usage and glutamine synthesis are highly activated in PDAC to support nucleotide syntheses and anabolic pathways critical for tumor growth [108,109]. With the shortage of raw materials, PDAC enhances autophagy and micropinocytosis mechanisms to reuse cellular and extracellular components to provide materials for other activities [110,111]. Through enhanced autophagy, CAFs release non-essential amino acids (mostly alanine) through exosomes to fuel the TCA cycle to support anabolic needs or promote the generation of Acetyl-CoA from glutamine [111,112]. CAFs also release lysophosphatidylcholine (LPC) which then is converted to lysophosphatidic acid (LPA) and exerted as mitogenic signals [113]. Due to polymorphisms in cellular and non-cellular components, PDACs are metabolically heterogeneous. However, since the metabolic program contributes significantly to neoplastic progression and tumor properties, there is a compelling rationale to use metabolic pathways as targets for developing PDAC therapeutics.

### 8.3. Immunological Transformation

PDAC is generally considered an immunologically ‘cold’ cancer because of its ability to (i) avoid immune detection, (ii) dampen anti-tumor immunity, and (iii) accumulate an immunosuppressive compartment. The immigration of effector immune cells toward cancer cells is firstly altered by desmoplastic stroma secreted by CAFs, which acts as a physical barrier and reduces blood perfusion. The recognition of tumor associated antigens (TAAs) is also limited by reduced expression of HLA class I on cancer cell membrane [114,115]. Furthermore, tumor cells upregulate the expression of HLA-related molecules (MICA/MICB) to inhibit NK and γδT cell responses to abnormal HLA expressing cells [101]. Concurrently, PDAC cells enhance the expression of PD-L1 to inhibit cytotoxic T lymphocyte activity [116]. Tumor cell-derived chemokines and pro-inflammatory cytokines secreted by CAFs recruit a variety of immune cells into the TME. Those immune cells firstly establish an inflammatory condition and then, through interplays with the TME components, establish an immunosuppressive milieu that supports cancer growth. The high concentration of lactate in TME induces T and NK cell activation and promotes immunosuppressive polarization [117]. The presence of tumor-derived colony-stimulating factor-1 (CSF-1) and IL-13 in TME polarizes the TAM from the pro-inflammatory M1 subtype toward the immunosuppressive M2 macrophage subtype [118]. Altogether, the PDAC TME includes diverse immune cells and secreted molecules which are oriented toward an immunosuppressive profile that mediate the crosstalk between these secreted factors and other TME compartments.

### 8.4. TME and the Metastatic Process

Metastasis in PDAC may occur at the earliest stages of PanIN formation due to (i) high invasiveness of tumor cells, (ii) metastatic support from TME, (iii) mechanisms to establish the pro-metastatic niche, and (iv) colonization and outgrowth at the distant organs.

In the very early stage, TGF-β secreted by both cancer cells and CAFs shapes the TME to promote tumor growth. First, HA and collagens in the dense stroma increase the detachment of cancer cells from the basement membrane and invasion to the surrounding stroma [119,120]. The biochemical and mechanical stimuli in ECM also promote cancer cell migration and invasion through various pathways [120]. Moreover, the hypoxic and inflammatory condition in PDAC TME induces the epithelial-to-mesenchymal transition (EMT). During the EMT, the epithelial cells acquire migratory and invasive properties of mesenchymal cells [121]. Additionally, the establishment of the tumor microenvironment of metastasis (TMEMs) is comprised of infiltrated macrophages and epithelial cells that induce cathepsin protease. This disrupts the E-cadherin junction and promotes vascular leakiness, ultimately leading to the intravasation of the tumor cells into the bloodstream [122,123].

The pro-metastasis niche is the conditioned environment in the host distant organ which attracts and supports cancer cell seeding, triggering the colonization and outgrowth of the secondary tumor. The most common distant organ for PDAC metastasis is the liver, followed by the lung [6]. However, in rarer cases, the PDAC cells can also present an adaptation to a varied range of organ microenvironments to form a tumor in the brain, bones or skin [6]. In general, the host organ responds to inflammatory signals from the primary tumor site through specific paracrine signals which may differ between organs but result in (i) increasing the presence of myeloid cells, including macrophages and neutrophils, and (ii) increasing the deposition of ECM proteins, such as collagen and fibronectin. Particularly, in liver metastasis, pancreatic stromal cells produce and release IL-6 into the portal vein and trigger hepatocyte activation from their resting stage [124]. In response to IL-6, hepatocytes activate STAT3 signaling, which eventually releases myeloid chemoattractants [124]. Furthermore, tumor-derived exosomes instruct liver resident macrophages to release TGF-β to make fibronectin that supports the recruitment of inflammatory cells [124]. Within the host organ, migratory cancer cells must succeed in establishing colonization or micro-metastatic dormancy, whereas the cancer cell increases the adhesive junctions and communicates with the surrounding niche to stimulate proliferation. To do so, the niche with seeded neoplastic cells need to recruit and accumulate hematopoietic cells (macrophages, neutrophils), CAFs, and epithelial cells to establish the mesenchymal-to-epithelial transition (MET). This micrometastasis is the foundation for a macrometastasis which, over time, forms a secondary tumor in the distant organ.

## 9. PDAC Therapeutics and Tumor Microenvironment

**Figure 2:**
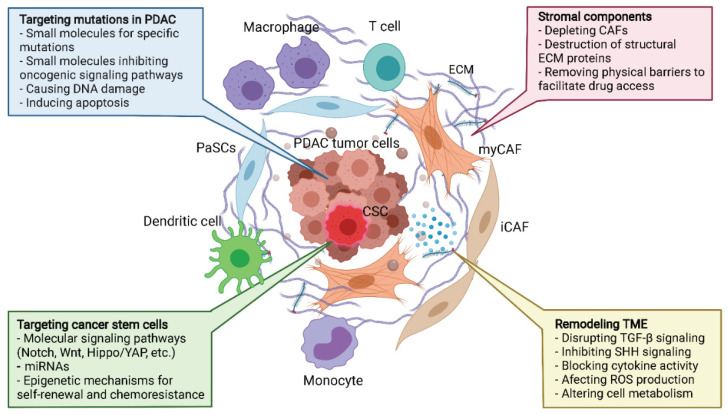
Neoplastic cells and ECM targeting approaches in PDAC treatment. Because of its large contribution to tumor development and treatment resistance, the heterogeneity of tumor cells, mutations, and ECM of PDAC are also attractive targets for therapeutic development. Targeting the KRAS protein and its upstream and downstream signaling are some of the most potential targets for drug development and are under investigation in several trials. Besides that, the therapeutic approaches for eliminating cancer stem cells in PDAC focus on targeting particular major signaling pathways by small molecules or targeting core genetic regulators, such as microRNA, as listed in the boxes. The ECM consists of collagens, fibronectin, and other proteins that can be targeted, together with other therapeutic strategies. PaSC = pancreatic stellate cells, PDAC = pancreatic ductal adenocarcinoma, EMC = extracellular matrix, myCAF = myofibroplastic cancer associated fibroblast, iCAF = inflammatory cancer associated fibroblast. Figure made in BioRender.

Over the past few decades, the effectiveness of PDAC treatments in the clinic have not yet improved significantly, but the accumulation of understanding in PDAC biology is opening up novel paradigms for therapeutic strategies that involve targeting the TME components (Figure 2).

### 9.1. Targeting Hallmark Mutations and Pancreatic Cancer Stem Cells

KRAS oncogenic activation is the most common mutation in PDACs that is present in most cancers and drives tumorigenesis by crosstalking to inflammatory pathways. Therefore, reducing KRAS activity or its up/downstream signaling is an attractive strategy. Novel inhibitors, such as AMG 510 and ARS1620, which directly target the KRAS G12C mutant variant, show a promising outcome, but this mutation only accounts for 1–4% of PDACs [125,126]. Another strategy is to target the most frequent KRAS mutation G12D by other mechanisms, such as siRNA [127,128]. The oncogenic KRAS properties are activated by Epidermal growth factor receptor (EGFR) signaling. Therefore, many trials using EGFR inhibitors plus standard gemcitabine for PDAC are ongoing and present encouraging outcomes in overall survival. Downstream signaling of KRAS, such as PI3K-AKT-mTOR and RAF-MEK-ERK, are also potential targets for drug development [129] and are currently under investigation in several trials.

CSCs refer to the subset of cancer cells within PDACs that have self-renewal capacity and are able to produce differentiated progeny. CSCs have been discovered in many cancer types, including in PDAC [130,131]. Pancreatic CSCs have an important role in PDAC growth, metastasis [130], chemoresistance in PDAC, and tumor recurrence following therapeutic treatment [132], as well as tumor stroma differentiation [133]. Therefore, the elimination of CSCs is a prerequisite for a successful therapy for curing PDAC.

Several strategies have been implemented to target CSCs, including targeting specific surface markers of CSCs, such as CD44, CD24, CD133, CXCR4, and EPCAM. For example, generating a bi-specific antibody against CSC surface markers EPCAM and CD3 has been shown to lead to CSC elimination by cytotoxic T lymphocytes in vitro and in vivo [134]. However, CSCs are not a distinct subpopulation of cancer cells but, rather, a dynamic state that non-CSC tumor cells are also able to enter, suggesting that there is plasticity in cellular identity involving differentiation and dedifferentiation processes [135]. Hence, a successful therapeutic strategy must take into account the elimination of existing CSCs, while also inhibiting molecular pathways that allow for cellular plasticity and the formation of new CSCs.

Multiple signaling cascades which involve the regulation of stem cell factors, cell cycle and epigenetic mechanisms that govern Pancreatic cancer stem cell (PaCSC) formation and maintenance of their stem cell-like properties are potential targets for drug development. The notable pathways that are under-evaluated now for PDAC treatment are Notch, Wnt, and Hedgehog because of their essential function in pancreatic embryonic development [136,137]. Other important pathways include Hippo/YAP, AKT/mTOR/PI3K, and TGF-β/Nodal/Activin signaling. The treatment outcomes could be improved significantly when combining the standard chemotherapy and small molecule compounds targeting particular signaling or epigenetic mechanisms that control gene expression of cancer stem cells. The Notch inhibitors PF-03084014, alone or in the combination with gemcitabine, showed a significantly inhibited PaCSCs and induced tumor regression in xenograft models [138]. Targeting the FZD receptor in the Wnt signaling by a monoclonal antibody, Vantictumab, in combination with nab-paclitaxel and gemcitabine, has been evaluated in several trials. As far as the role of Nodal/Activin signaling has been revealed, in PDAC tumorigenesis, as well as PaCSCs regulation, inhibition of Nodal signaling could be a potential therapeutic target for drug development. More recently, there has been increasing evidence demonstrating that microRNAs (miRNAs) play a crucial role in Regulating PaCSCs [139]. The dysregulated expression of several miRNAs is associated with gemcitabine-resistance, such as miR-146, -205, -7, etc. [139]. Although a lot of effort is still needed for understanding the regulation of PaCSCs, the new insight in this field will facilitate more efficient therapies to treat PDAC.

### 9.2. Targeting the Stroma—The Physical Barrier to Therapeutics

The dense stroma plays an important role in tumor progression, drug resistance and metastasis in PDAC, making it a potential therapeutic target for single or combined strategies. The approach to targeting the stromal desmoplasia could involve (i) inhibition of signaling, such as SHH, that is responsible for the development of stroma, (ii) elimination of ECM depositing cells, such as CAFs, or (iii) disruption of the ECM structural components, such as collagen or HA. Despite the solid evidence for molecular mechanisms and success in preclinical trials, these strategies have not brought the outcomes for PDAC patients but have shown unexpected results instead.

The combination of SHH inhibitors, such as vismodegib, with gemcitabine not only did not show improvement in overall survival but also led to a higher rate in disease progression [140]. Small molecular inhibitors, such as UAMS 1110 or talabostat, which selectively deplete CAFs, together with the standard treatment of gemcitabine, did not present a promising outcome in patients [141,142]. Furthermore, the genetic deletion of alpha-smooth muscle actin CAFs in a mouse model even led to a more aggressive phenotype of tumor cells [40]. To disrupt the ECM, HA or collagen have been targeted by altering the MMPs or proteolytic enzymes, such as the human recombinant PH20 hyaluronidase (PEGPH20) [143,144,145], but those trials did not yield clinical benefits. The failures in those clinical trials may be due to several factors, including (i) the drugs/molecules have high toxicity in humans, resulting in an unsustainable duration of treatment to get significant outcome, (ii) the particular stromal component may play both anti-tumor and pro-tumor properties which are highly dynamic over time, and (iii) there are other pathways in the TME that need to be targeted simultaneously with the stromal targets.

### 9.3. Immunotherapy in PDAC Treatment

#### 9.3.1. Cancer Vaccine

**Figure 3:**
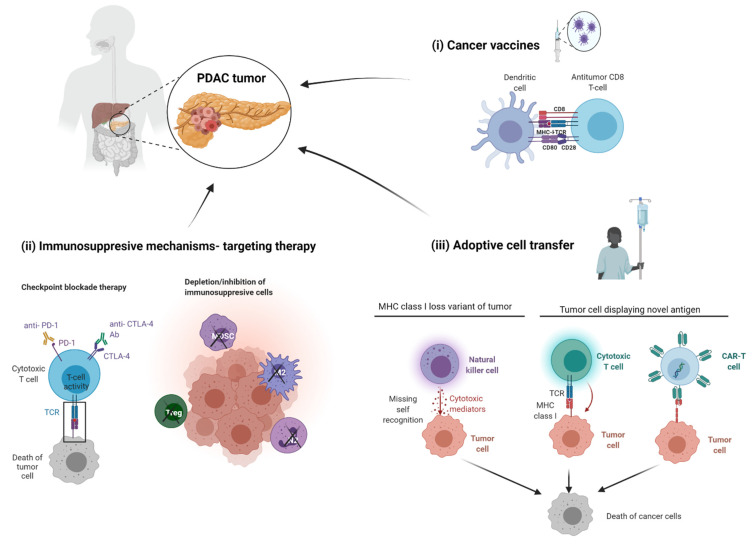
Immunotherapies to treat PDAC. There are several proposed strategies to target immunological aspects of PDAC which are (**i**) cancer vaccines, (**ii**) targeting immunosuppressive mechanism by immune checkpoint blockage therapy, and (**iii**) adoptive cell transfer which are comprised of NK, Cytotoxic lymphocyte, and CAR-T therapies. PDAC = pancreatic ductal adenocarcinoma, PD-1 = programmed cell death protein 1, CTLA-4 = cytotoxic T lymphocyte associated protein 4, TCR = T cell receptor, Treg = regulatory T cell, MDSC = myeloid derived suppressor cell, M2 = Tumor associated M2 macrophage, N2 = tumor associated N1 neutrophil, MHC = major histocompatibility complex, CAR = chimeric antigen receptor. Figure made in BioRender.

PDACs can evade immunity in many ways, and each process can be investigated for therapeutic development (Figure 3). First, focusing on the limitation of cancer antigen presentation in PDAC, cancer vaccine strategies have been investigated, including peptide-based vaccine, virus-based vaccine, DNA-based vaccine, or cell-based vaccine. However, despite the positive effects recorded in phases I and II, some vaccine strategies, such as Telovac, PANVAC, or algenpantucel-L, failed in phase III [55]. Notably, the trial with GVAX, a tumor cell vaccine transfected with the granulocyte–macrophage colony-stimulating factor (GM-CSF) gene, showed efficacy in mobilizing effector immune cells into the TME. Therefore, treatment strategies in combination with GVAX are still ongoing [49]. The above results suggest that vaccination alone is not sufficient to achieve effective anti-tumor immunity in PDAC.

#### 9.3.2. Targeting the Immunosuppressive Mechanisms

PDACs are typically characterized as immunologically ‘cold’ tumors, suggesting that they do not elicit active anti-tumor immune responses, whereas genomic analysis of PDACs has revealed that only a subtype of PDACs is immunologically active [146,147]. The immunological ‘coldness’ of PDACs involves multiple mechanisms. This includes an immunosuppressive microenvironment with the expression of PD-L1 and IDO1, as well as a low tumor mutation burden that would otherwise initiate neoantigenicity [49,70,148,149]. Additionally, a dense desmoplastic stroma acts as a physical barrier to immune cell infiltration and is a contributing factor to the lack of success of immunotherapy in PDAC and limited responses of PDACs treated with immune-checkpoint inhibitors [150,151]. The cellular and acellular constituents involved in immunosuppressive mechanisms are potential therapeutic targets. These strategies include directly targeting cells, such as Tregs, or targeting cytokines/chemokines that recruit them to TME. Tregs are one of the most attractive candidates which can be selectively targeted by low-dose of cyclophosphamide [152]. Treg depletion by cyclophosphamide, together with GVAX, shows an augmented immune response to PDAC [153]. Other strategies, such as depleting TAM M2 macrophages by CSF1/CSF1R blockade, are under investigation [154].

As with many solid tumors, immune checkpoint inhibitors (ICIs) have been tested in PDAC treatment in many clinical trials with the most universal targets being CTLA-4 and PD1/PD-L1. The ICIs therapies are using monoclonal antibodies against CTLA4, such as ipilimumab, tremelimumab, or PD1, such as pembrolizumab, durvalumab, or BMS-956559, together with or without other chemotherapy agents. Almost all of those trials of ICI therapy for PDAC treatment have only published data for safety and dose escalation, while opening the opportunity to further studies for evaluating efficacy.

A distinct approach focusing on immunosuppressive TME is to target the co-stimulatory molecules present on antigen presenting cell (APC), such as DCs and M1 macrophages. The combination of anti-CD40 monoclonal antibody and gemcitabine with/without anti-PD-1 Ab shows promising outcomes [155]. In the overall strategy to target the stroma-immune cell crosstalk, the hope is to simultaneously eliminate not just CAFs but also impact the immune cells in the TME.

### 9.4. Adoptive Cell Transfer

Adoptive cell transfer (ACT) therapy is an approach of infusing autologous or allogeneic immune cells for enhancing the specific recognition, targeting, and elimination of tumor cells in patients. Two main strategies in ACT therapy are (i) increasing the number of effector cells, such as TILs, NK, iNKT cells, and (ii) manipulating the anti-tumor properties of a particular lymphocyte population. The latter is a universal approach that uses genetically-modified T cells for expressing the artificially designed chimeric antigen receptor (CAR), which will specifically recognize tumor cells and activate the elimination mechanism. The well-known PDAC tumor-associated antigens, such as CEA, EGFR, Her2, MUC1, and mesothelin, are targeted for CAR-T development [156]. Recently, a team led by John F. Marshall developed and demonstrated the clinical potential of the carcinoembryonic antigen-related cell adhesion molecule 7 (CEACAM7)—a novel target for CAR-T development for PDAC treatment with promising outcomes [157]. Pre-conditioning strategies, such as optimizing the TH2-TH1 ratio, depletion of Tregs, and increasing macrophage activation for enhancing CAR-T cell therapy, have also been investigated [156]. The toxicity of specific molecular target, together with the immunosuppressive properties of PDAC TME, are the main obstacles in developing CAR-T cell therapy for PDAC treatment.

### 9.5. Remodeling TME—The Global Approach

Besides the aforementioned approaches, which have yielded both promising and unexpected clinical outcomes, PDAC TME’s unique properties are still valuable for exploiting alternative therapeutic strategies. While the paradigm to target exclusively the stromal or immunological aspect of PDAC has provided limited benefits in patients, efforts which concentrate on remodeling PDAC TME by multiple approaches might be favorable. Several outstanding PDAC TME remodeling strategies include targeting TGF-β signaling, blocking cytokine activity and signaling, such as SHH, inducing ROS production, and targeting metabolic mechanisms. Trials in large cohorts are being conducted to evaluate the effectiveness of incorporating TME remodeling strategies with standard chemotherapy using gemcitabine or FOLFIRINOX. It is likely that a combination of different strategies targeting cancer stem cells, the bulk of tumor cells, as well as components of TME, will provide improved results in clinical trials. Therefore, more studies are urgently needed to bring us closer to more effective therapies that benefit PDAC patients.

## 10. Conclusions

The TME plays an important role in the initial PanIN stages, tumor growth, metastasis, and drug resistance of PDAC. Therefore, it is necessary to elucidate the cellular and molecular mechanisms of PDAC TME to develop effective clinical therapeutics. TME in PDAC shows high heterogeneity and is comprised of cancerous cells, immune cells, and CAFs, as well as acellular constituents. Moreover, the interplay of those cellular populations and acellular factors creates a dynamic TME from the early stages of PanINs and induces drug resistance, early metastasis, and disease recurrence in PDAC patients. Although there has been an accumulation of new knowledge in PDAC biology over the past few decades, there are still no effective treatments for PDAC. Further studies need to refine the crosstalk between cancer cells, including the regulation of pancreatic cancer stem cells, CAFs, and immune cells in PDAC TME. Besides that, the translational approaches should focus on a combination of therapeutic targets that are aimed at multiple features of PDAC TME, rather than focus solely on a particular aspect. Although many trials have recorded failures in clinical outcomes, we have never had as much understanding of the biology of PDAC as we do now, and it is an important foundation for developing effective therapies in the future.

## Abbreviations

APC	Antigen presenting cell
BM-MSC	bone marrow mesenchymal stem cell
CAF	cancer-associated fibroblast
CAR-T	chimeric antigen receptor T-cell
CCL	C-C Motif Chemokine Ligand
CD	cluster of differentiation
CEA	carcinoembryonic antigen
CEACAM7	carcinoembryonic antigen-related cell adhesion molecule 7
CSC	cancer stem cells
CSF	colony-stimulating factor
CTLA-4	cytotoxic T lymphocyte associated protein 4
CXCL	chemokine (C-X-C motif) ligand
ECM	extracellular matrix; EGFR: epidermal growth factor receptor
EMT	epithelial-mesenchymal transition
FAP	fibroblast activation protein
GEMM	genetically engineered mouse models
GVAX	granulocyte-macrophage colony-stimulating factor gene transduced autologous pancreatic cancer vaccine
HGF	hepatocyte growth factor
HIF:	hypoxia-inducible factor
iCAFs	inflammatory CAFs
ICI	immune checkpoint inhibitors
IL	interleukin
JAK-STAT	Janus kinase-signal transducer and activator of transcription
LIF	Leukemia Inhibitory Factor
MDSC	myeloid-derived suppressor cells
MET	mesenchymal to epithelial transition
MHC	major histocompatibility complex
MMP	matrix metalloproteinase
myCAFs	myofibroblastic CAFs
NE	neutrophil elastase
NF-kB	nuclear factor kappa-light-chain-enhancer of activated B cells
NK	natural killer
PaCSC	pancreatic cancer stem cell
PanIN	pancreatic intraepithelial neoplasia
PaSC	pancreatic stellate cell
PD-1	programmed cell death protein 1
PDAC	pancreatic ductal adenocarcinoma
PEGPH20	human recombinant PH20 hyaluronidase
RNS	reactive nitrogen species
ROS	reactive oxygen species
SHH	sonic hedgehog
SPARC	secreted protein acidic and rich in cysteine
TAM	tumor-associated macrophages
Teff	effector T cell
TGF-β	transforming growth factor beta
TME	tumor microenvironment
TAN	tumor-associated neutrophils
TNF-α	tumor Necrosis Factor alpha
Treg	regulatory T cells
α-SMA	alpha smooth muscle actin
